# Asymmetric deep cerebral venous filling predicts poor outcome of acute basilar artery occlusion after endovascular treatment

**DOI:** 10.1111/cns.14513

**Published:** 2023-11-12

**Authors:** Huiyuan Wang, Panpan Shen, Xinyue Yu, Yafei Shang, Jie Xu, Xinyi Chen, Mingming Tan, Longting Lin, Mark Parsons, Sheng Zhang, Yu Geng

**Affiliations:** ^1^ Center for Rehabilitation Medicine, Department of Neurology Zhejiang Provincial People's Hospital (Affiliated People's Hospital, Hangzhou Medical College) Hangzhou China; ^2^ School of Clinical Medicine, Graduate School Bengbu Medical College Bengbu China; ^3^ The Second Clinical Medical College Zhejiang Traditional Chinese Medicine University Hangzhou China; ^4^ Alberta Institute Wenzhou Medical University Wenzhou China; ^5^ Department of Quality Management Zhejiang Provincial People's Hospital Hangzhou China; ^6^ School of Medicine and Public Health University of Newcastle New South Wales Newcastle Australia; ^7^ Department of Neurology, Liverpool Hospital University of New South Wales New South Wales Sydney Australia

**Keywords:** basilar artery ischemia, cerebral edemas, cerebral veins, prognosis, thrombectomy

## Abstract

**Objective:**

To explore the relationship between asymmetric deep cerebral venous (ADCV) filling and poor outcomes after endovascular treatment (EVT) in patients with acute basilar artery occlusion (ABAO).

**Methods:**

ABAO patients were selected from a prospectively collected data at our center. The DCV filling was evaluated using computed tomography perfusion (CTP)‐derived reconstructed 4D‐DSA or mean venous map. ADCV filling was defined as the internal cerebral vein (ICV), thalamostriate vein (TSV), or basal vein of Rosenthal (BVR) presence of ipsilateral filling defects or delayed opacification compared to the contralateral side. Poor prognosis was defined as a modified Rankin scale score >3 at the 90‐day follow‐up.

**Results:**

A total of 90 patients were enrolled in the study, with a median Glasgow Coma Scale of 6, 46 (51.1%) showed ADCV filling, 59 (65.6%) had a poor prognosis, and 27 (30.7%) had malignant cerebellar edema (MCE). Multivariate adjusted analysis revealed significant associations between asymmetric TSV and poor prognosis (odds ratio, 9.091, *p* = 0.006); asymmetric BVR (OR, 9.232, *p* = 0.001) and asymmetric ICV (OR, 4.028, *p* = 0.041) were significantly associated with MCE.

**Conclusion:**

Preoperative ADCV filling is an independent influencing factor for the poor outcome after EVT in ABAO patients.

## Graphical abstract

In patients with acute basilar artery occlusion with asymmetric deep cerebral venous filling, the risk of postoperative malignant cerebellar edema and adverse outcome at 90‐day follow‐up is significantly increased even after complete recanalization.

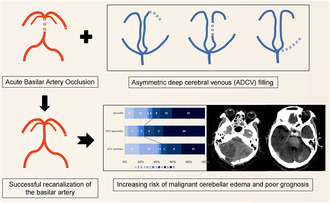

## 1 INTRODUCTION

Acute basilar artery occlusion (ABAO) is a subtype of stroke with high mortality and severe disability rates,^1^ particularly in the absence of successful recanalization. Recent multicenter prospective studies have demonstrated that early endovascular treatment (EVT) can reduce patient mortality and improve clinical outcomes.^2^ However, despite successful recanalization, ABAO patients still experience high rates of postoperative malignant cerebellar edema (MCE) and ineffective reperfusion, leading to significant mortality and disability.^2^ Due to differences in posterior circulation anatomy and post‐occlusion manifestations compared to anterior circulation stroke, factors influencing the prognosis of EVT in anterior circulation stroke may not necessarily apply to ABAO patients. Therefore, it is crucial to explore factors that affect adverse outcomes after EVT in ABAO patients to improve neurological functional recovery.

Currently, most imaging studies on adverse outcomes of EVT in ABAO patients have focused primarily on arterial collateral circulation, such as the relationship between the Basilar Artery on Computed Tomography Angiography Prognostic (BATMAN) Score,^3^ the posterior circulation collateral score (PC‐CS),^4^ and prognosis. Very few studies have investigated the role of the venous system in ABAO. However, in anterior circulation large‐vessel occlusive stroke (ALVOS), venous filling has been shown to be significantly associated with outcomes after EVT and has become a research focus. In ALVOS patients, poor preoperative cortical venous opacification^5^, ^6^, ^7^ is significantly associated with postoperative malignant brain edema and poor prognosis, while good preoperative venous filling also increases the EVT benefit in patients with a large core infarct volume.^8^ Additionally, comprehensive venous assessment, by Adusumilli et al.,^9^ has demonstrated the important role of internal cerebral veins (ICV) filling in EVT patients of the anterior circulation. Given the anatomical differences between the posterior and anterior circulation veins, intracranial deep venous drainage may play a crucial role in ABAO patients.^10^ Moreover, the location of thalamostriate veins (TSV), ICV, and the basal vein of Rosenthal (BVR) is relatively fixed and easily identifiable by clinicians.

Therefore, the objective of our study is to explore the correlation between admission asymmetric deep cerebral venous (ADCV) filling and EVT outcomes in ABAO patients and to explore the occurrence mechanism. We hypothesized that ADCV filling is associated with poor outcome of EVT.

## 2 METHODS

### 2.1 Study population

We analyzed data from ABAO patients who underwent EVT at Zhejiang Provincial People's Hospital between October 2016 and February 2023, collected as part of our prospective continuous study. Inclusion criteria were as follows: (1) age between 18 and 80 years; (2) absence of intracranial hemorrhage on initial cranial computed tomography (CT); (3) confirmation of basilar artery occlusion by computed tomographic angiography (CTA) and digital subtraction angiography (DSA); (4) within 24 hours of disease onset and had received EVT; (5) baseline National Institutes of Health Stroke Scale (NIHSS) score >6; (6) accepted computed tomography perfusion (CTP) imaging pre‐EVT. Exclusion criteria were as follows: (1) significant cerebellar infarction with evident edema on admission cranial CT scan; (2) presence of severe comorbidities with an expected survival of less than 90 days; (3) lack of pre‐EVT CTP or poor imaging quality; (4) lack of follow‐up data after EVT. All patients or their appropriate family member signed written informed consent before EVT.

**FIGURE 1:**
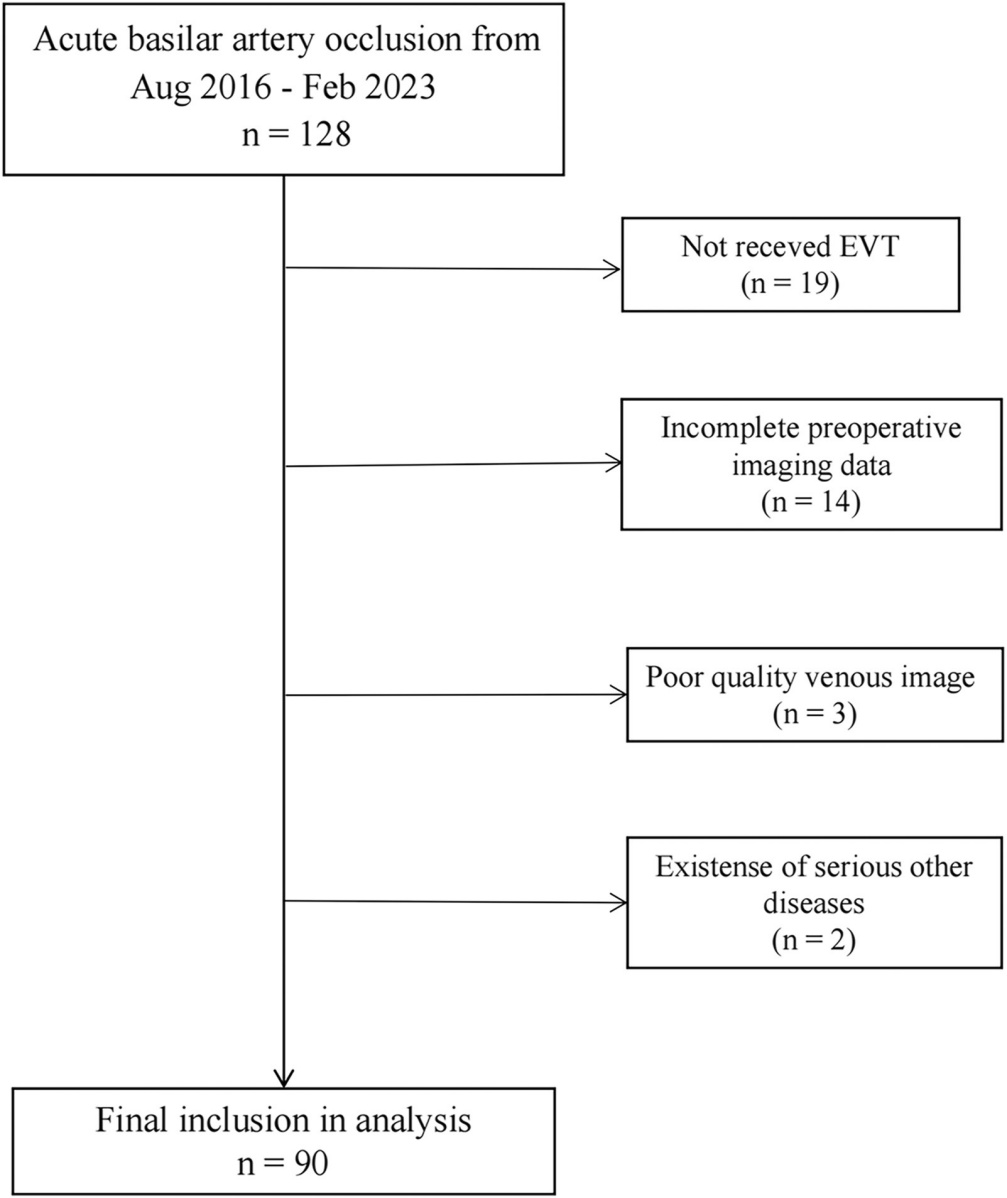
Flowchart of patient selection. EVT, endovascular treatment.

The study was approved by the Medical Ethics Committee of Zhejiang Provincial People's Hospital (NO.2019KY050). All study protocol were conducted according to the principles expressed in the Declaration of Helsinki. Figure 1 shows the screening flow of the patients in this study.

### 2.2 Data collection

We collected demographic information, including age, gender, and medical history such as hypertension, diabetes, and atrial fibrillation, as well as a prior history of stroke. Clinical information included the baseline NIHSS score, GCS score, intravenous thrombolysis, baseline blood pressure, the time from onset to puncture (OTP), and the time from puncture to recanalization (TPR). Laboratory data included baseline blood glucose, neutrophil count, hemoglobin, and platelet count. Imaging data comprised the admission pc‐ASPECT score, the Basilar Artery on Computed Tomography Angiography Prognostic (BATMAN) score, asymmetric deep venous filling, and postoperative follow‐up findings of hemorrhage and edema on NCCT. Additionally, we collected the modified Rankin Scale (mRS) score at 3 months post‐EVT. Poor prognosis was defined as an mRS score >3 at 90 days after EVT.

### 2.3 Imaging protocols

All patients underwent baseline CTP and noncontrast CT (NCCT) scans using the Toshiba Aquilion 320‐layer CT scanner and commercial software (MIStar; Apollo Medical Imaging, Melbourne, Australia) and acquired four‐dimensional (4D) CTA images and mean venous images. The pc‐ASPECT and BATMAN scores, as well as postoperative imaging data, were evaluated by two blinded neuroradiologists (Xu and Chen).

Malignant cerebellar edema (MCE) was defined as extensive cerebellar swelling with evident mass effect observed on postoperative follow‐up CT scans.

### 2.4 Definition of asymmetric intracranial deep vein filling

**FIGURE 2:**
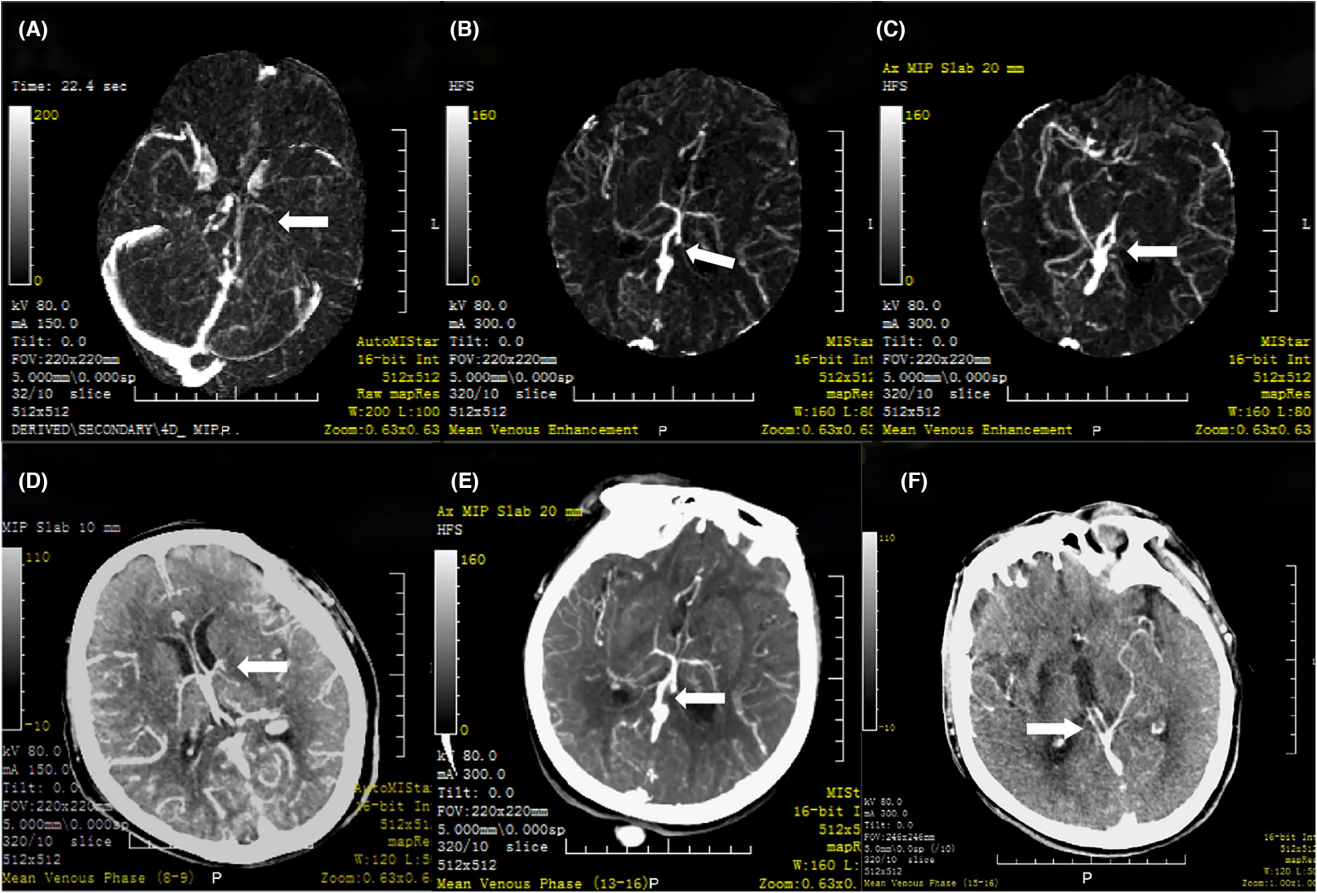
Observation and evaluation of deep cerebral venous filling. Deep cerebral venous filling can be observed on both the reconstructed 4D‐DSA and the mean venous map derived from CT perfusion software. (A–C) represent the asymmetric thalamostriate vein (TSV), internal cerebral vein (ICV), and basal vein of Rosenthal (BVR) observed on the reconstructed 4D‐DSA map, while (D–F) represent the asymmetric TSV, ICV, and BVR observed on the mean venous map.

The filling of ICV, TSV, and BVR was assessed using reconstructed 4D‐DSA and mean venous map derived from MIStar software. ADCV was defined as delayed filling, significant thinning, or complete absence of filling on ipsilateral compared to the contralateral side. Figure 2 illustrates the observed ADCV filling on both reconstructed 4D‐DSA and mean venous map. The venous images were evaluated collectively by two neuroradiologists (Shen and Shang) who were blinded to other imaging and clinical data, and an independent observer (Zhang), who was trained in vein assessment, provided additional evaluation.

### 2.5 Statistical analysis

Continuous variables were described as mean ± standard deviation (SD) or median (25th, 75th percentile), while categorical variables were presented as frequencies (percentages). The reliability of vein assessment among observers was assessed using the Kappa statistic. Prior to commencing statistical analyses, we performed a normality assessment on all continuous data through the utilization of the Kolmogorov–Smirnov test. Subsequently, for data that did not adhere to the normal distribution assumption, we utilized pertinent non‐parametric tests for subsequent analysis. Group comparisons were performed using the Student's *t*‐test or Mann–Whitney U test for continuous variables and the Pearson chi‐square test or Fisher's accurate test for categorical data. Variables with a *p*‐value less than 0.05 in the univariate analysis were included in the binary logistic regression analysis, recorded odds ratios (ORs) and 95% confidence intervals (CIs), and receiver operating characteristic (ROC) curve analysis was used to evaluate the discriminatory ability of ADCV for predicting MCE and poor prognosis. *p* value of <0.05 was considered statistically significant.

Statistical analysis was conducted using IBM SPSS Statistics software (version 26.0; IBM Corporation).

## 3 RESULTS

**FIGURE 3:**
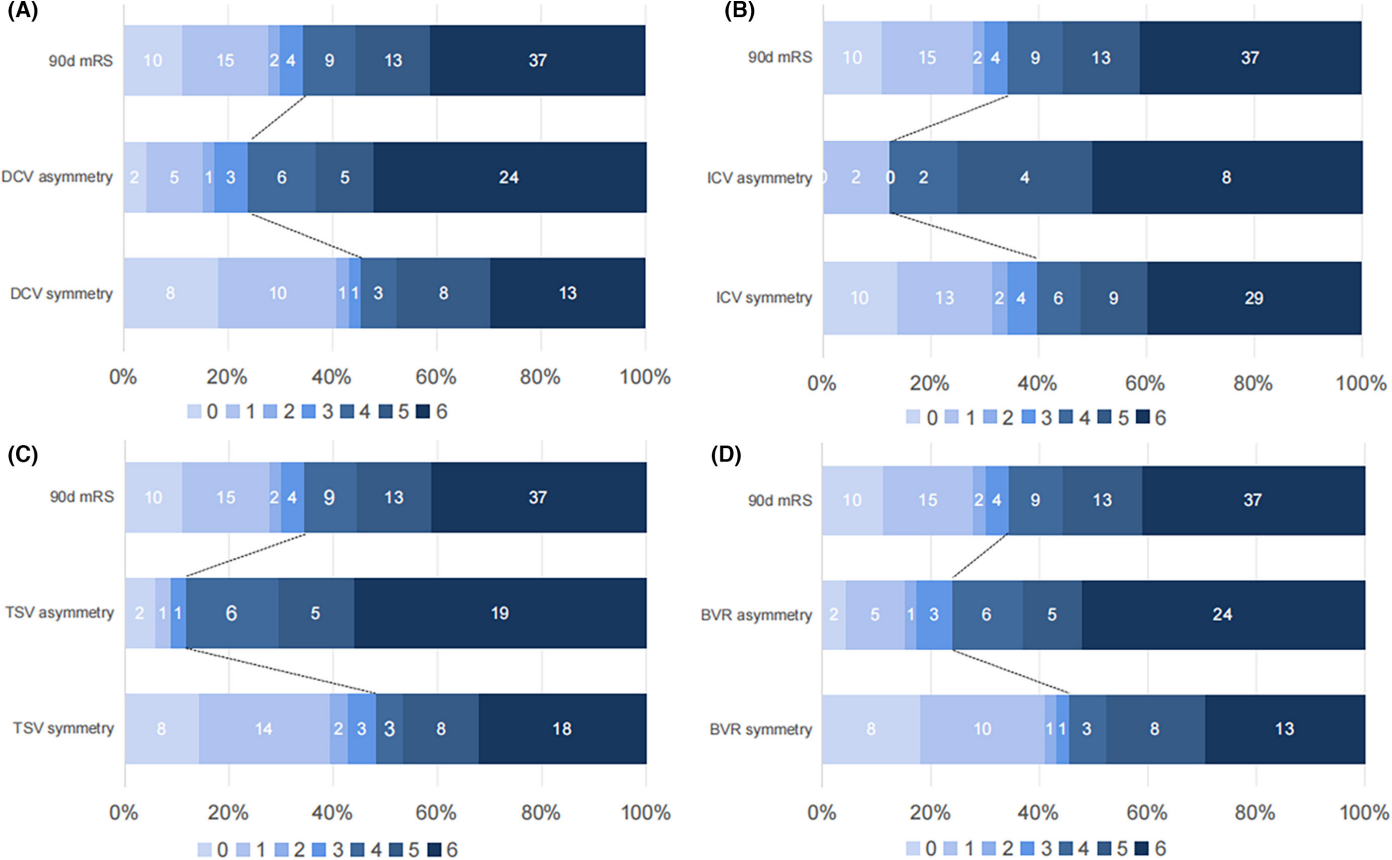
Distribution of mRS at 90‐d follow‐up after EVT in ABAO patients. Asymmetric DCV indicates at least one pair of asymmetric deep cerebral venous filling. mRS, modified Rankin Scale. ICV, Internal Cerebral Vein; TSV, Thalamostriate Vein; BVR, Basal Vein of Rosenthal.

In our study, 90 ABAO patients were enrolled, with two excluded from MCE analysis due to lack of follow‐up imaging in critically ill postoperative states. Median age was 66 years (55–75), with 29 cases (32.2%) being female. Median GCS score was 6, and poor prognosis was observed in 59 cases (46.9%). Among the 88 patients with complete imaging data, 27 cases (9.2%) had MCE. In 46 (51.1%) of the 90 patients, ADCV filling was observed. Among these cases, 34 (37.8%) had asymmetric TSV, 46 (51.1%) had asymmetric BVR, and 16 (17.7%) had asymmetric ICV. In patients with ADCV filling, 37 (80.4%) had a poor prognosis, while it occurred in 22 (50%) of patients with good DCV filling (*p* = 0.002). Figure 3 illustrates the distribution of mRS scores at the 90‐day follow‐up in ABAO and ADCV patients. Table 1 shows the baseline characteristics of the patients, revealing that ADCV had a higher occurrence rate in hypertensive patients (*p* = 0.003). Furthermore, a further analysis of the different veins was conducted. It was found that there is a correlation between higher baseline blood glucose and asymmetric BVR (*p* = 0.001), as well as a higher baseline neutrophil count in patients with asymmetric TSV (*p* = 0.048). However, the relationship between ADCV and the thrombus location in ABAO patients did not reach statistical significance (*p* = 0.245), and the proportion of asymmetric TSV was higher in patients with tandem occlusions but did not reach statistical significance (*p* = 0.064).

**TABLE 1:** Patient baseline data and results of univariate analysis of poor prognosis.

Variables	Total	Symmetric DCV	Asymmetric DCV	*p* value
Age, year, median (IQR)	66 (55, 75)	60 (52, 76)	67 (60, 73)	0.121
Female, *n* (%)	29 (32.2)	13(29.5)	16 (34.8)	0.595
Hypertension, *n* (%)	57 (63.3)	15 (47.7)	36 (78.3)	0.003**
Atrial fibrillation, *n* (%)	23 (25.6)	13 (29.5)	10 (21.7)	0.396
Diabetes, *n* (%)	19 (21.1)	7 (15.9)	12 (26.1)	0.237
Stroke/TIA, *n* (%)	21 (23.3)	12 (27.3)	9 (19.6)	0.387
Antiplatelet, *n* (%)	26 (28.9)	15 (34.1)	11 (23.9)	0.287
Stroke characteristics				
Baseline NIHSS score, median (IQR)	30 (19, 35)	30 (15, 36)	31 (21, 34)	0.887
GCS, median (IQR)	6 (4, 11)	6 (4, 13)	6 (4, 10)	0.546
pc‐ASPECTS, median (IQR)	7 (6, 8)	7 (6, 8)	7 (5, 8)	0.668
Baseline SBP, mmHg, average (SD)	159.50 (24.57)	158.73 (25.38)	160.24 (24.04)	0.772
Baseline DBP, mmHg, average (SD)	89.13 (15.42)	91.16 (17.51)	87.20 (13.02)	0.228
Glucose, mg/dL, median (IQR)	7.46 (6.52, 11.11)	7.45 (4.91, 9.82)	7.94 (7.21, 10.46)	0.104
Neutrophils, (×10^9^/L), median (IQR)	8.23 (5.84, 9.95)	6.72 (5.51, 9.28)	10.78 (8.96, 12.11)	0.071
Hemoglobin, (g/L), average (SD)	141.33 (20.53)	141.86 (22.55)	140.83 (18.64)	0.812
Platelet (×10^9^/L), average (SD)	193.59 (61.20)	201.23 (67.57)	186.28 (54.16)	0.249
Intravenous thrombolysis	33 (36.7)	18 (40.9)	15 (32.6)	0.414
BATMAN, median (IQR)	5 (4,7)	5 (4,7)	5 (4,7)	0.451
OTP, min, median (IQR)	445 (280, 691)	385 (283, 644)	513(267, 828)	0.202
PTR, min, median (IQR)	60 (41, 117)	59 (40, 117)	66 (45, 115)	0.508
mTICI 2b‐3, *n* (%)	82 (91.1)	39 (88.6)	43 (93.4)	0.286
Cause, *n* (%)				
Cardioembolic	27 (30.0)	14 (31.8)	13 (28.3)	
Large artery atherosclerosis	50 (55.6)	23 (52.3)	27 (58.7)	
Others	13 (14.4)	7 (16)	6 (13.0)	
Postintervention characteristics				
MCE, *n* (%)	27 (29.7)	9 (20.5)	18 (39.1)	0.037*
Poor prognosis	59 (65.6)	22 (50)	37 (80.7)	0.002**
Total, *n*	90	44	46	

*Note*: Values presented are number (percentage) for categorical and median (interquartile range) or average (SD, standard deviation) for ordinal and continuous variables. Good prognosis, 90‐d mRS score 0–3; poor prognosis, 90‐d mRS score 4–6.

Abbreviations: BATMAN, basilar artery on computed tomography angiography; DBP, diastolic blood pressure; DCV, deep cerebral venous; GCS, Glasgow Coma Scale; MCE, Malignant cerebellar edema; mRS, modified Rankin Scale; mTICI, modified Treatment in Cerebral Infarction; NIHSS, National Institutes of Health Stroke Scale; OTP, the time from onset to puncture; pc‐ASPECTS, posterior circulation Alberta Stroke Program Early CT Score; PTR, time from puncture to recanalization; SBP, systolic blood pressure.

Table 2 presents the results of univariate analysis for MCE, while Table 3 provides the results of univariate analysis for prognosis in ABAO patients.

**TABLE 2:** Univariate analysis of the malignant cerebellar edema.

Variables	Non‐MCE	MCE	*p* value
Age, year, median (IQR)	70 (54, 78)	64 (57, 68)	0.094
Female, *n* (%)	22 (36.6)	6 (22.2)	0.210
Hypertension, *n* (%)	37 (61.7)	19 (70.4)	0.159
Atrial fibrillation, *n* (%)	18 (30.0)	5 (18.5)	0.279
Diabetes, *n* (%)	11 (18.3)	7 (25.9)	0.397
Stroke/TIA, *n* (%)	15 (25.0)	6 (22.2)	0.810
Antiplatelet, *n* (%)	20 (33.3)	6 (22.2)	0.316
Stroke characteristics			
Baseline NIHSS score, median (IQR)	30 (14, 35)	31 (24, 35)	0.196
GCS, median (IQR)	6 (4, 13)	6 (4, 9)	0.223
pc‐ASPECTS, median (IQR)	7 (6, 9)	7 (5, 8)	0.210
Baseline SBP, mmHg, average (SD)	159.87 (25.21)	157.33 (23.39)	0.658
Baseline DBP, mmHg, average (SD)	89.33 (16.07)	88.59 (14.55)	0.839
Glucose, mg/dL, median (IQR)	7.35 (6.30, 8.70)	9.01 (7.20, 12.06)	0.011*
Neutrophils, (×10^9^/L), median (IQR)	7.52 (5.14, 9.32)	9.80 (8.26, 11.20)	<0.001**
Hemoglobin, (g/L), average (SD)	136.90 (21.33)	151.41 (15.36)	0.002**
Platelet (×10^9^/L), average (SD)	191.75 (61.44)	195.89 (63.14)	0.773
Intravenous thrombolysis	19 (31.6)	13 (48.1)	0.126
BATMAN, median (IQR)	5 (4, 7)	5 (4, 6)	0.159
ICV asymmetry	6 (10.0)	10 (37.0)	0.002**
TSV asymmetry	20 (33.3)	12 (44.4)	0.294
BVR asymmetry	22 (36.6)	23 (85.2)	<0.001**
OTP, min, median (IQR)	425 (284, 674)	502 (254, 773)	0.750
PTR, min, median (IQR)	60 (40, 116)	60 (42, 113)	0.869
mTICI 2b‐3, *n* (%)	55 (91.7)	26 (96.3)	0.530
Total, n	61	27	

*Note*: Values presented are number (percentage) for categorical and median (interquartile range) or average (SD, standard deviation) for ordinal and continuous variables.

Abbreviations: BATMAN, basilar artery on computed tomography angiography; BVR, Basal Vein of Rosenthal; DBP, diastolic blood pressure; DCV, deep cerebral venous; ICV, Internal Cerebral Vein; GCS, Glasgow Coma Scale; MCE, Malignant cerebellar edema; mTICI, modified Treatment in Cerebral Infarction; NIHSS, National Institutes of Health Stroke Scale; OTP, the time from onset to puncture; pc‐ASPECTS, posterior circulation Alberta Stroke Program Early CT Score; PTR, time from puncture to recanalization; SBP, systolic blood pressure; TSV, Thalamostriate Vein.

*p values indicate *p* < 0.05 and **p values indicate *p* < 0.01.

**TABLE 3:** Univariate analysis of poor prognosis.

Variables	Good prognosis	Poor prognosis	*p* value
Age, year, median (IQR)	60 (51, 76)	66 (57, 74)	0.361
Female, *n* (%)	10 (32.2)	19 (32.2)	0.996
Hypertension, *n* (%)	15 (48.4)	42 (71.2)	0.033*
Atrial fibrillation, *n* (%)	9 (29.0)	14 (23.7)	0.584
Diabetes, *n* (%)	1 (3.2)	18 (30.5)	0.002*
Stroke/TIA, *n* (%)	5 (16.1)	16 (27.1)	0.241
Antiplatelet, *n* (%)	11 (35.5)	15 (25.4)	0.317
Baseline NIHSS score, median (IQR)	18 (11, 28)	34 (26, 36)	<0.001**
GCS, median (IQR)	12 (6, 13)	6 (4, 8)	<0.001**
pc‐ASPECTS, median (IQR)	8 (6, 9)	6 (5, 8)	0.022*
Baseline SBP, mmHg, average (SD)	150.71 (20.64)	164.12 (25.36)	0.013*
Baseline DBP, mmHg, average (SD)	89.35 (15.96)	89.02 (15.27)	0.923
Glucose, mg/dL, median (IQR)	7.07 (6.25, 8.50)	7.76 (6.7, 10.94)	0.036*
Neutrophils, (10^9^/L), median (IQR)	6.72 (5.51, 9.28)	8.75 (6.21, 10.27)	0.012*
Intravenous thrombolysis	11 (36.4)	22 (37.3)	0.886
Hemoglobin, (g/L), average (SD)	139.39 (21.60)	142.36 (20.10)	0.518
Platelet (×10^9^/L), average (SD)	197.87 (62.34)	191.34 (61.01)	0.633
BATMAN, median (IQR)	6 (5, 7)	4 (4, 6)	0.055
Asymmetric ICV	2 (6.5)	14 (23.7)	0.035*
Asymmetric TSV	4 (12.9)	30 (50.8)	<0.001**
Asymmetric BVR	11 (35.4)	35 (59.3)	0.020*
OTP, min, median (IQR)	410 (265, 632)	461(280, 773)	0.305
PTR, min, median (IQR)	65 (40, 103)	60 (42, 120)	0.777
mTICI 2b‐3, *n* (%)	28 (90.3)	54 (91.5)	0.876
MCE, *n* (%)	1 (3.2)	26 (44.0)	<0.001**
Total, *n*	31	59	

*Note*: Values presented are number (percentage) for categorical and median (interquartile range) or average (SD, standard deviation) for ordinal and continuous variables.

Abbreviations: BATMAN, basilar artery on computed tomography angiography; BVR, Basal Vein of Rosenthal; DBP, diastolic blood pressure; GCS, Glasgow Coma Scale; ICV, Internal Cerebral Vein; mTICI, modified Treatment in Cerebral Infarction; NIHSS, National Institutes of Health Stroke Scale; OTP, the time from onset to puncture; pc‐ASPECTS, posterior circulation Alberta Stroke Program Early CT Score; PTR, time from puncture to recanalization; SBP, systolic blood pressure; TSV, Thalamostriate Vein.

*p values indicate *p* < 0.05 and **p values indicate *p* < 0.01.

### 3.1 Association between ADCV and MCE

**FIGURE 4:**
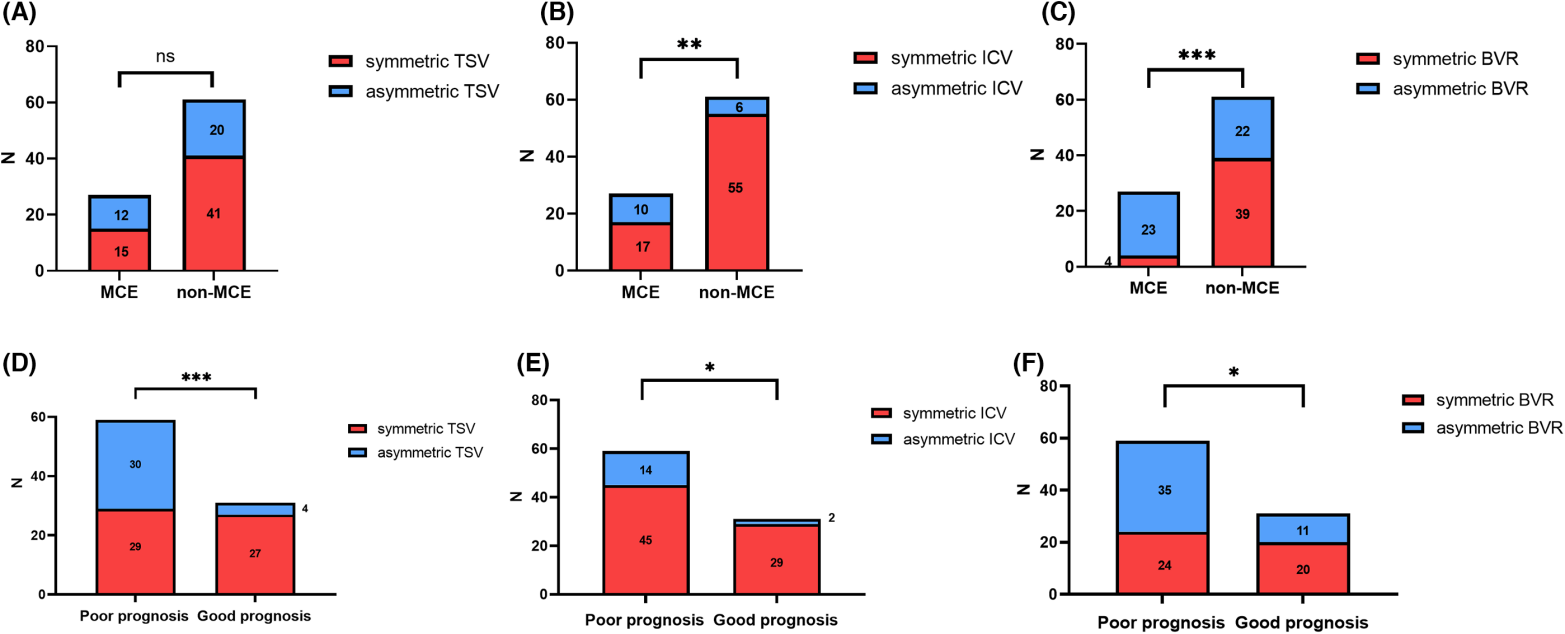
Chi‐square tests revealed a significant association between asymmetric deep cerebral venous filling and poor outcomes following EVT. MCE, Malignant cerebellar edema. Poor prognosis, 90‐d mRS score 4–6; mRS, modified Rankin Scale. TSV, Thalamostriate Vein; ICV, Internal Cerebral Vein; BVR, Basal Vein of Rosenthal. *Indicate *p* < 0.05, **Indicate *p* < 0.01 and ***Means *p* < 0.001.

**FIGURE 5:**
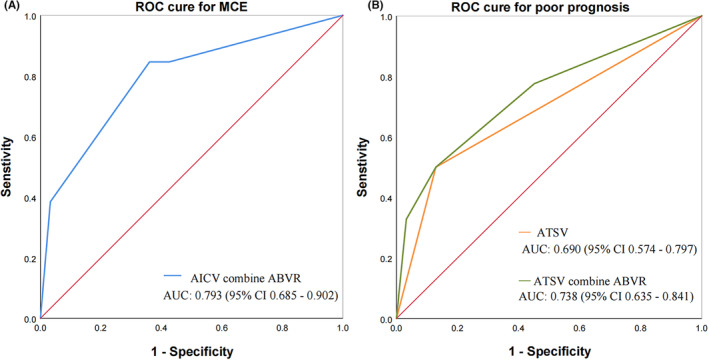
ROC curve for asymmetric deep cerebral venous filling predicts poor outcomes following EVT. A represents the ROC curve for the combined index of asymmetric ICV and BVR in predicting MCE, while B represents the ROC curves for asymmetric TSV (orange curve) and the combined index of asymmetric TSV and BVR (green curve) in predicting poor prognosis after EVT.

**FIGURE 6:**
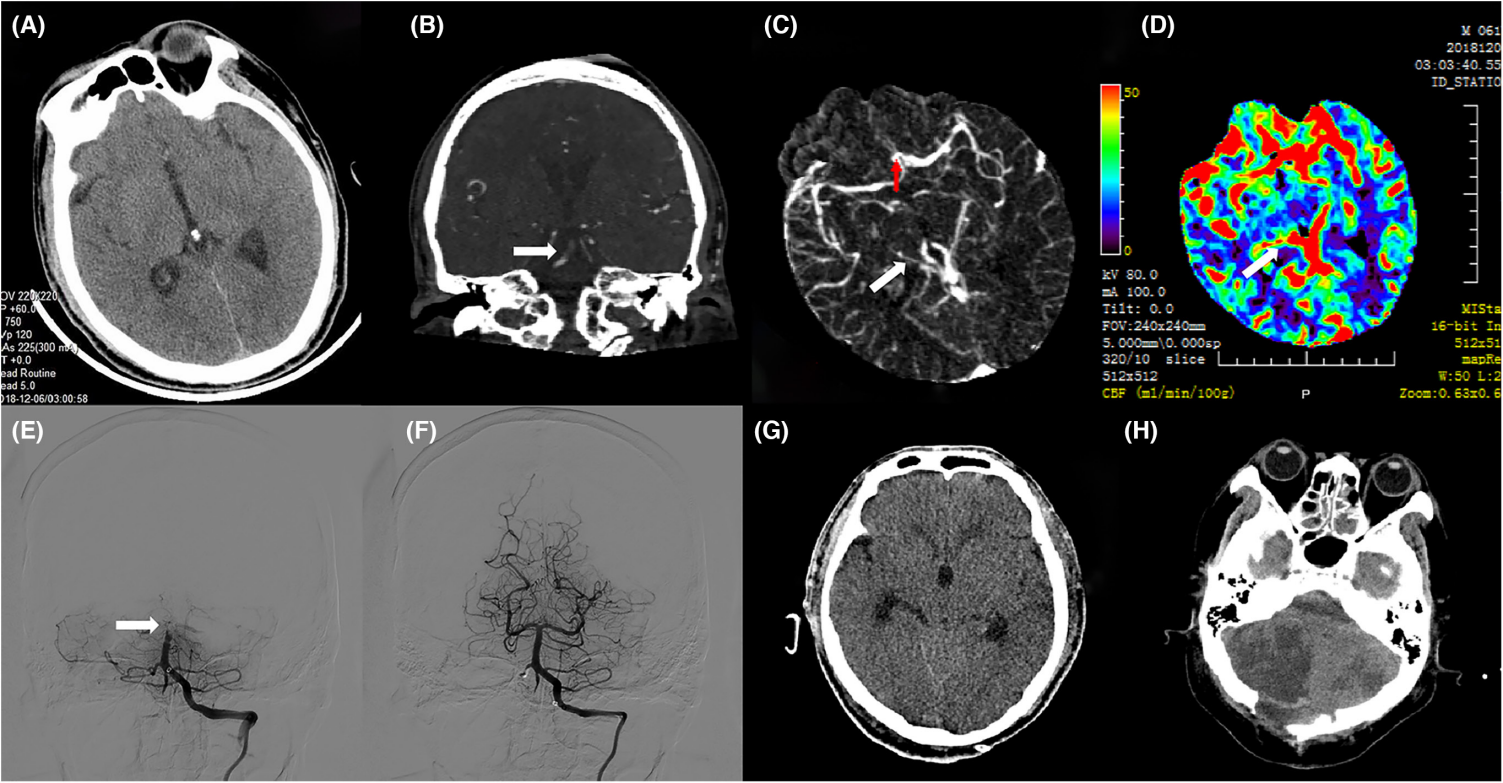
Example for illustrating the associations between asymmetric BVR and malignant cerebellar edema after EVT. A patient presented with acute basilar artery occlusion (ABAO), as indicated by the white arrow on computed tomographic angiography (CTA, Figure B) and digital subtraction angiography (DSA, Figure E), preoperative brain CT revealed no hemorrhage (Figure A). The patient exhibited asymmetric basal vein of Rosenthal (BVR) on 4D‐CTA (Figure C, white arrow) and on cerebral blood flow (CBF) map (Figure D, white arrow). Endovascular treatment (EVT) achieved complete recanalization of the basilar artery (Figure F). However, a 24 h noncontrast CT (NCCT) scan after EVT revealed massive edema of the cerebellum with mass effect (Figure G), leading to subsequent decompressive craniectomy (Figure H).

In the univariate analysis of postoperative MCE, apart from baseline blood glucose, neutrophil count, asymmetric ICV (*p* = 0.002), and asymmetric BVR (*p* < 0.001) were significantly associated with MCE following EVT. Figure 4A–C illustrates the correlation between ADCV and MCE. After adjusting for multiple factors in the regression analysis (Table 4), admission neutrophil count, asymmetric ICV (OR = 4.028, 95% CI: 1.062–15.276; *p* = 0.041), and asymmetric BVR (OR = 9.232, 95% CI: 2.421–35.199; *p* = 0.001) were identified as independent risk factors for MCE. The ROC curve analysis (Figure 5A) revealed that the combined measure of asymmetric ICV and asymmetric TSV predicted post‐EVT MCE with an area under the curve (AUC) of 0.793 (95% CI: 0.685–0.902), with a maximum Youden index sensitivity of 84.6% and specificity of 63.9%. Figure 6 explains the occurrence of MCE in an ABAO patient with asymmetric BVR despite achieving reperfusion of the basal artery through EVT.

**TABLE 4:** Multivariate regression analysis of the adverse outcomes after EVT.

Variables	OR	95% CI	*p* value
Poor prognosis			
pc‐ASPECTS	0.679	0.466–0.988	0.043*
Baseline NIHSS	1.169	1.084–1.260	<0.001**
TSV asymmetry	9.091	1.869–44.228	0.006**
BVR asymmetry	4.645	1.210–17.837	0.025*
MCE			
Baseline neutrophils	1.372	1.104–1.705	0.004*
ICV asymmetry	4.028	1.062–15.276	0.041*
BVR asymmetry	9.232	2.421–35.199	0.001**

Abbreviations: BVR, Basal Vein of Rosenthal; CI, confidence interval; ICV, Internal Cerebral Vein; MCE, Malignant cerebellar edema; NIHSS, National Institutes of Health Stroke Scale; OR, odds ratio; pc‐ASPECTS, posterior circulation Alberta Stroke Program Early CT Score; TSV, Thalamostriate Vein.

*p < 0.05, **p < 0.01 of the multivariate regression analysis.

### 3.2 Association between ADCV and poor prognosis

In the univariate analysis, a significant association was observed between a history of hypertension, a history of diabetes, baseline GCS, NIHSS, BATMAN score, SBP, and poor prognosis, and there is a significant association between ADCV and poor prognosis (*p* = 0.002). Figure 4D–F demonstrates the correlation between ADCV filling and poor prognosis, with significant associations found for asymmetric TSV (*p* < 0.001), asymmetric BVR (*p* = 0.020), and asymmetric ICV (*p* = 0.035). After adjusting for multiple factors in the regression analysis (Table 4), pc‐ASPECTS, baseline NIHSS, asymmetric TSV (OR = 9.091, 95% CI: 1.869–44.228; *p* = 0.006), and asymmetric BVR (OR = 4.645, 95% CI: 1.210–17.837; *p* = 0.025) were identified as independent risk factors for adverse outcomes at 90 days following EVT.

In order to further investigate the relationship between ADCV and MCE as well as poor prognosis, we included TSV, BVR, and MCE as variables in a logistic regression analysis of poor prognosis. The results demonstrated that even after adjusting for MCE, asymmetric TSV remained significantly associated with adverse outcomes (OR = 7.210, 95% CI: 2.058–25.253; *p* = 0.002). On the other hand, there was no statistical difference observed for asymmetric BVR (*p* = 0.870), indicating that the impact of asymmetric BVR on poor prognosis is mediated through MCE. The combined measure of asymmetric TSV and asymmetric BVR for predicting poor prognosis in the ROC curve analysis (Figure 5B) demonstrated that although the addition of asymmetric BVR did not significantly improve the discriminatory ability for poor prognosis, the combined measure showed a certain degree of predictive value for poor prognosis (AUC = 0.738, 95% CI: 0.635–0.841). However, its predictive capability was lower than that of the combined measure of ADCV for predicting MCE.

## 4 DISCUSSION

Our study elucidates that the ADCV filling, inclusive of TSV, ICV, and BVR, independently influences the prognosis of ABAO patients subjected to EVT. ADCV filling is correlated with an increased risk of adverse prognosis among this patient cohort. These insights advance ABAO patient EVT strategies, underscoring venous drainage's pivotal role in treatment outcomes and aligning with established knowledge of veins' significance in anterior circulation large‐vessel occlusive strokes.

The study also explored other factors influencing adverse outcomes after EVT. pc‐ASPECTS^11^, ^12^ was associated with poor prognosis at 90‐day follow‐up, while admission neutrophil count^13^, ^14^ was an independent factor for MCE, consistent with previous studies.

Currently, dynamic CTA or susceptibility‐weighted imaging (SWI) are commonly used methods for venous evaluation in acute ischemic stroke patients.^15^, ^16^ SWI has advantages in assessing intracranial deep venous imaging, but it is challenging to apply for baseline venous evaluation in ABAO patients. TSV, ICV, and BVR have relatively fixed positions with minimal variability, making them easily identifiable on dynamic CTA with speed and accuracy. Considering the anatomical factors of cerebral venous drainage,^10^ while superficial venous has received more attention in venous studies of anterior circulation EVT patients, the DCV may play a more significant role in ABAO patients.

TSV is the largest venous branch of the ICV and primarily drains the basal ganglia region and deep cerebral structures, which is often considered a landmark for the ventricles in neurosurgical procedures.^17^ The ICV receives drainage from the TSV as well as the lateral ventricle, thalamus, and lateral veins of the brainstem before joining the BVR. The BVR is an important component of the deep veins,^17^ not only receiving drainage from the ICV but also receiving venous drainage from the temporal lobe, midbrain, and pons, ultimately merging with the Galen vein. In our study, the BVR demonstrated significant clinical potential and was significantly associated with MCE after EVT, possibly because asymmetric BVR filling implies a larger extent of affected venous branches.

Our study supports the association between ADCV filling and hypertension in ABAO patients. Cao et al.^18^ noted that elevated admission systolic (≥140 mmHg) and diastolic (≥90 mmHg) blood pressures reduced reperfusion therapy success and increased adverse outcome risks in BAO patients, emphasizing blood pressure management's role in posterior circulation stroke. The impact of hypertension on microcirculation has been extensively studied in the cardiovascular research, revealing reduced distal vessel density and microcirculatory perfusion in hypertensive patients.^19^ Our study indicates that hypertensive BAO patients may have a higher likelihood of venous drainage obstruction than those with normal blood pressure.

Our study also observed a trend between neutrophil count and admission blood glucose levels and ADCV in ABAO patients. Previous studies have implicated the involvement of inflammatory responses in the pathophysiological mechanisms of cerebral venous thrombosis.^20^, ^21^ Inflammatory reactions occurring after ABAO can activate inflammatory cells, promote neutrophil adhesion, increase the release of inflammatory mediators, further exacerbate blood–brain barrier disruption, aggravate cerebral edema, and potentially lead to venous infarction.^20^ It is worth noting that our study did not find a relationship between thrombus location, arterial collaterals, and ADCV, which is consistent with venous studies in the ALVOS, possibly due to the autoregulatory mechanisms of blood flow between arteries and veins.^7^ However, given our small sample size, these findings may not be sufficient to draw definitive conclusions, and future larger‐scale prospective studies are needed to explore the factors influencing ADCV in ABAO patients.

The impact of ADCV filling on poor outcomes in ABAO patients primarily manifests as an increased risk of MCE and ineffective reperfusion, Where asymmetric BVR and ICV significantly increased the risk of MCE, while asymmetric TSV affected the neurological recovery after EVT. ADCV filling indicates the formation of microthrombi within the veins, leading to venous outflow obstruction,^5^ elevated venous pressure reduces cerebrospinal fluid drainage, resulting in intracranial hypertension.^22^ ICV and BVR have a broader drainage range, and when there is inadequate filling, they can affect the cerebrospinal fluid drainage. The degree of edema is more severe when ICV and BVR have inadequate filling compared to TSV. Simultaneously, the disrupted blood–brain barrier in ABAO patients activates inflammatory responses and releases pro‐inflammatory mediators, further exacerbating venous infarction and vasogenic brain edema.^23^, ^24^ Swollen brain tissue compresses draining veins, perpetuating a vicious cycle and contributing to the development of MCE.

Moreover, TSV drains the deep basal ganglia, compared to the other two veins, which is more reflective of the perfusion status of the microcirculation. In patients with ischemic stroke, the simultaneous reduction in cerebral arterial and venous blood flow signifies compromised microcirculatory flow between arteries and veins, leading to more severe cerebral ischemia and resulting in irreversible neuronal damage.^25^ Even with complete reperfusion of the basilar artery, achieving favorable clinical outcomes remains challenging.

Our study has several limitations. Firstly, due to the low incidence of ABAO, our Chinese single‐center study had a small sample size, which limited our ability to fully explore the predictive ability of ADCV for poor outcomes after EVT and investigate the factors influencing ADCV. Secondly, because the occurrence rate of intracranial hemorrhagic transformation in BAO patients is low, we did not assess the relationship between adverse venous filling and post‐EVT intracranial hemorrhagic transformation. We believe that future prospective multicenter studies can be conducted to investigate the factors influencing ADCV in BAO patients, explore the relationship between ADCV and intracranial hemorrhagic transformation, and develop a rational combined arterial and venous collateral scoring system to assist in selecting suitable ABAO patients for EVT. Thirdly, there is significant variability among cerebral veins. Our ADCV definition may introduce bias, as it could include patients with clearly slender congenital venous variations. However, our study included three pairs of veins that were relatively fixed in position and exhibited a reasonable degree of symmetry, thus significantly reducing this bias. Furthermore, exclusion criteria encompassed patients without prior CTA examination and those with inadequate image quality. Such exclusions could introduce selection bias and potentially influence study outcomes. Lastly, due to DSA limitations in vein observation, we chose CTA images and MIStar software to evaluate ADCV, which might moderately affect our assessment of ADCV. We believe that in future studies, further exploration of the consistency of other perfusion software for venous assessment can be conducted.

## 5 CONCLUSION

In conclusion, this study's findings indicate that ADCV undermines the benefits of EVT in ABAO patients and increases the risks of post‐EVT MCE and unfavorable outcomes at 90‐day follow‐up. These findings may assist clinicians in selecting ABAO patients who are likely to benefit from EVT.

## FUNDING INFORMATION

This study was supported by the Postgraduate Research Innovation Program of Bengbu Medical College (grant number: Byycx22116) and the Zhejiang Provincial Science and Technology Department Foundation (grant number: 2022C35071).

## CONFLICT OF INTEREST STATEMENT

The authors have no conflicts of interest to declare.

## PATIENT CONSENT STATEMENT

Informed consent was obtained from all individual participants included in the study.

## Data Availability

The data that support the findings of this study are available from the corresponding author upon reasonable request.
